# Managing children with daytime urinary incontinence: a survey of Dutch general practitioners

**DOI:** 10.1080/13814788.2022.2149731

**Published:** 2023-04-25

**Authors:** Antal P. Oldenhof, J. Marleen Linde, Ilse Hofmeester, Martijn G. Steffens, Francis J. Kloosterman-Eijgenraam, Marco H. Blanker

**Affiliations:** a Department of Urology, Isala Hospital, Zwolle, the Netherlands; b Department of General Practice and Elderly Care Medicine, University of Groningen, University Medical Centre Groningen, Groningen, the Netherlands; c Department of Paediatrics, Isala Hospital, Zwolle, the Netherlands

**Keywords:** Surveys, general practice/family medicine, urology, paediatrics

## Abstract

**Background:**

In the Netherlands, parents of children with daytime urinary incontinence (UI) first consult general practitioners (GPs). However, GPs need more specific guidelines for daytime UI management, resulting in care and referral decisions being made without clear guidance.

**Objectives:**

We aimed to identify Dutch GP considerations when treating and referring a child with daytime UI.

**Methods:**

We invited GPs who referred at least one child aged 4–18 years with daytime UI to secondary care. They were asked to complete a questionnaire about the referred child and the management of daytime UI in general.

**Results:**

Of 244 distributed questionnaires, 118 (48.4%) were returned by 94 GPs. Most reported taking a history and performing basic diagnostic tests like urine tests (61.0%) and physical examinations (49.2%) before referral. Treatment mostly involved lifestyle advice, with only 17.8% starting medication. Referrals were usually at the explicit wish of the child/parent (44.9%) or because of symptom persistence despite treatment (39.0%). GPs usually referred children to a paediatrician (*n* = 99, 83.9%), only referring to a urologist in specific situations. Almost half (41.4%) of the GPs did not feel competent to treat children with daytime UI and more than half (55.7%) wanted a clinical practice guideline. In the discussion, we explore the generalisability of our findings to other countries.

**Conclusion:**

GPs usually refer children with daytime UI to a paediatrician after a basic diagnostic assessment, usually without offering treatment. Parental or child demand is the primary stimulus for referral.

KEY MESSAGES

- Most Dutch GPs do not treat children with daytime urinary incontinence but refer them to a paediatrician
- Almost half of the GPs feel incompetent to treat children with DUI
- GPs need a general practice guideline in daily practice for DUI in children

## Introduction

Urinary incontinence (UI) is the involuntary leakage of urine from age 5 years or older [1]. Associated with shame, stress and social difficulties in children and parents alike [2–5], it affects self-confidence and impairs quality of life [5–7].

The treatment of UI in the Netherlands is multidisciplinary. Parents first seek help from a general practitioner (GP) or youth healthcare practitioner. If these physicians cannot resolve the problem, they can refer the child to a paediatrician or (paediatric) urologist. Although the Dutch associations of Urology and Paediatrics have collaborated to create guidance for assessing and treating daytime UI [8], no specific guideline exists for GPs. Studies in New Zealand and Australia showed that confidence in managing daytime UI seems to vary in primary care [9,10]. No comparable studies are available in the Netherlands and it is unclear how Dutch GPs approach daytime UI in children, how confident they are with this care, and on what basis they refer to secondary care. We aimed to identify these topics.

## Methods

### Study design

We performed a survey among GPs who referred children aged 4–18 years with daytime UI to the outpatient clinic of a large teaching hospital in the Netherlands between January 2018 and September 2019. We searched for cases based on Diagnosis Treatment Combination codes (DBC) recorded in secondary care medical charts and reviewed the referral letter to obtain the reason for referral.

Children referred for daytime UI, with or without coexisting nocturnal enuresis, were included. Monosymptomatic nocturnal enuresis and referral with urinary tract infections (UTIs) as the only cause for UI were exclusion criteria. General information, such as the child’s age and gender, was obtained from the referral letter or medical file. Finally, we invited the GPs of identified patients to participate in this survey.

### Survey

We constructed a questionnaire in a multidisciplinary team comprising a GP, a urologist, an epidemiologist and independent researchers based on clinical experiences, (inter)national guidelines and previous research [8,10]. To retrieve information on actual cases, the questionnaire included seven patient-specific questions. Additionally, eight general questions about the treatment of daytime UI were used (Appendix A).

GPs who referred more than one child were asked to complete the patient-specific information for each child and the general part only once. If a colleague referred a child, we asked the GP who received the questionnaire to respond on their colleague’s behalf.

The questionnaires were sent a maximum of 1 year after referral, and reminders were sent to GPs who had yet to respond after 2 weeks.

### Statistical analysis

Descriptive characteristics are reported for patient demographics and reason for referral, GP referral preferences, experience as a GP, interest in urological complaints, and self-rated skill in treating daytime UI in children.

Normality was assessed using the Kolmogorov–Smirnov test. Medians and interquartile ranges (IQR) are reported for non-normally distributed data. Categorical variables are presented as percentages and compared with the chi-square test. We calculated 95% confidence intervals for some categorical variables. Possible correlations between different ordinal variables are shown using the Spearman rank correlation coefficient (*r_s_*), considering a *p*-value < 0.05 to be statistically significant. Correlation is shown graphically by an interpolation line between the known values, offering a simplified view of the relationship. The data were analysed using IBM SPSS, Version 25.0 (IBM Corp., Armonk, NY).

### Ethics

The Medical Ethical Committee of Isala Zwolle confirmed that formal ethical approval was not necessary under the Dutch law.

## Results

### Participants

Of 201 children referred to urologists and 959 referred to paediatricians, 25 and 219 met the inclusion criteria, respectively.

In total, 94 GPs returned 118 questionnaires (1–4 per GP), with the data for 96 cases (81.4%) completed by the referring GP. Seven GPs ended only the patient-specific part without answering the general questions, while five GPs did not respond to the general part. Complete data were available for 72 unique GPs, 40 females (55.6%) and 32 males (44.4%), with a median working experience of 11.5 years (IQR, 13.3 years).

The 118 children included 63 (53.4%) males, with a median age of 6 years (IQR, 4 years) (Table 1).

**Table 1.** Demographic characteristics of the children.

	*Total*	*Referral*	
		*Urology*	*Paediatrics*
	*n =* 118	*n =* 19	*n =* 99
Male, *n* (%)	63 (53.4)	8 (42.1)	55 (55.6)
Female, *n* (%)	55 (46.6)	11 (57.9)	44 (44.4)
Age (in years), median, [IQR]	6 [4]	9 [8]	6 [4]

*Abbreviations*: IQR, interquartile range.

#### Management of daytime UI

##### Discussed complaints

Most GPs discussed the coexistence of nocturnal enuresis (73.7%), whether UI was primary or secondary (68.8%), the defaecation pattern (63.6%) and/or micturition habits (61.9%) (Table 2). GPs less frequently asked if coexisting pain (43.2%), mental problems, social problems or UTIs were present. Eight GPs (6.8%) did not discuss any complaints and referred directly to the hospital.

**Table 2.** Patient-specific questions, diagnostics *(n = 118).*

Focus of question	Response*	*n* (%)
Discussed complaints	None	8 (6.8)
	Nocturnal enuresis	87 (73.7)
	Primary or secondary UI	81 (68.6)
	Defaecation pattern	75 (63.6)
	Micturition habits	73 (61.9)
	Pain	51 (43.2)
	Other	18 (15.3)
Diagnostics used	None	27 (22.9)
	Physical examination	58 (49.2)
	Genital investigation	31 (26.3)
	Urine test (dipstick or microscopy)	72 (61.0)
	Urine culture	23 (19.5)
	Voiding diary (incl. fluid intake)	13 (11.0)
	Ultrasound kidneys	0
	Other	5 (4.2)

*Multiple answers possible per GP.

*Abbreviations*: UI, urinary incontinence.

##### Diagnostics

Overall, 22.9% of GPs performed no diagnostics, 49.2% performed a physical examination and 26.3% inspected the genital area (Table 2). In 61.0% of cases, urine was checked by dipstick or microscopy and followed by a urine culture in 19.5%. A voiding diary was advocated by 11% of GPs. Reasons for not performing diagnostics were that parents wanted a referral or that diagnostics had already been done at a prior referral or by a physical therapist.

##### Lifestyle advice

More than half of the GPs (61.9%) gave lifestyle advice (Table 3), including the need for sufficient fluid intake (34.7%), adequate toilet posture and hygiene (34.7%), a high-fibre diet (29.7%) and having set voiding times (28.0%).

**Table 3.** Used therapy by general practitioners before referral because of daytime urinary incontinence and reasons for referral (*n* = 118).

Focus of question	Response*	*n* (%)
Lifestyle advice	None	45 (38.1)
	Sufficient fluid intake	41 (34.7)
	High-fibre food	35 (29.7)
	Adequate toilet posture and hygiene	41 (34.7)
	Voiding at set time	33 (28.0)
	Other	11 (9.3)
Medical therapy	None	97 (82.2)
	Anticholinergics	1 (0.8)
	Laxatives	17 (14.4)
	Other	4 (3.4)
Other therapies	Pelvic floor therapy	13 (11.0)
	Other	16 (13.6)
Reason for referral	Explicit wish patient/parent(s)	53 (44.9)
	Too little experience/knowledge	19 (16.1)
	Persistent symptoms	46 (39.0)
	Other	36 (30.5)

*Multiple answers possible per GP.

##### Treatment

Some GPs (17.8%) started pharmaceutical treatment (Table 3), most commonly laxatives (14.4%). Only one GP started anticholinergics, two had used desmopressin. Most GPs (80.0%) did not think that treatment with anticholinergic drugs was appropriate for primary care. GPs referred to a pelvic floor physiotherapist in 11.0% of cases.

##### Referral

Most common reasons for referral were the explicit wish of a parent or patient (44.9%) or the persistence of symptoms despite treatment (39.0%) (Table 3).

Most children were referred to paediatricians (83.9%), which most GPs reported as their preference (72.9%). Sometimes there was a desire for a more general approach, especially in cases with coexisting constipation, behavioural problems or other comorbidities. Arguments cited for referral to a urologist were parental request, the presence of an anatomic abnormality and recommendation by a pelvic floor therapist.

##### Competence and interest

**Figure 1.**
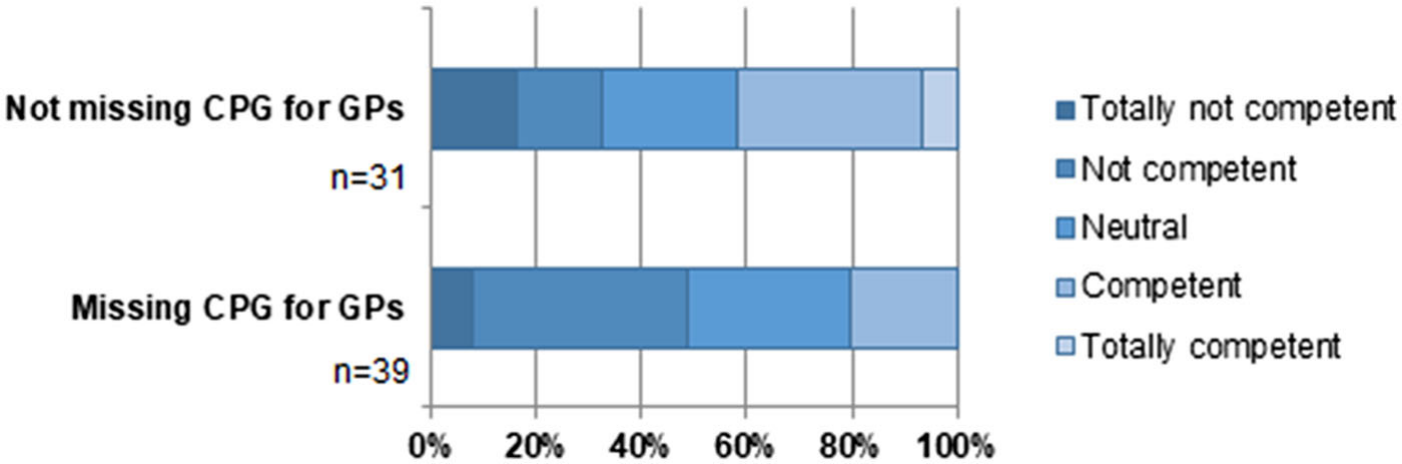
Perceived skill in treating daytime urinary incontinence (%) of GPs by whether they want a CPG for daytime urinary incontinence. *Abbreviations*: CPG, clinical practice guideline; GPs, general practitioners.

Of the GPs, 41.4% felt incompetent in treating children with UI and 30% felt (totally) competent. More than half (55.7%) stated they wanted a clinical practice guideline for GPs (Figure 1). Some GPs consulted guidelines on nocturnal enuresis or recurrent UTIs (*n* = 12) or the guideline from Dutch associations of Urology and Paediatrics (*n* = 2), but most GPs did not use a guideline.

**Figure 2.**
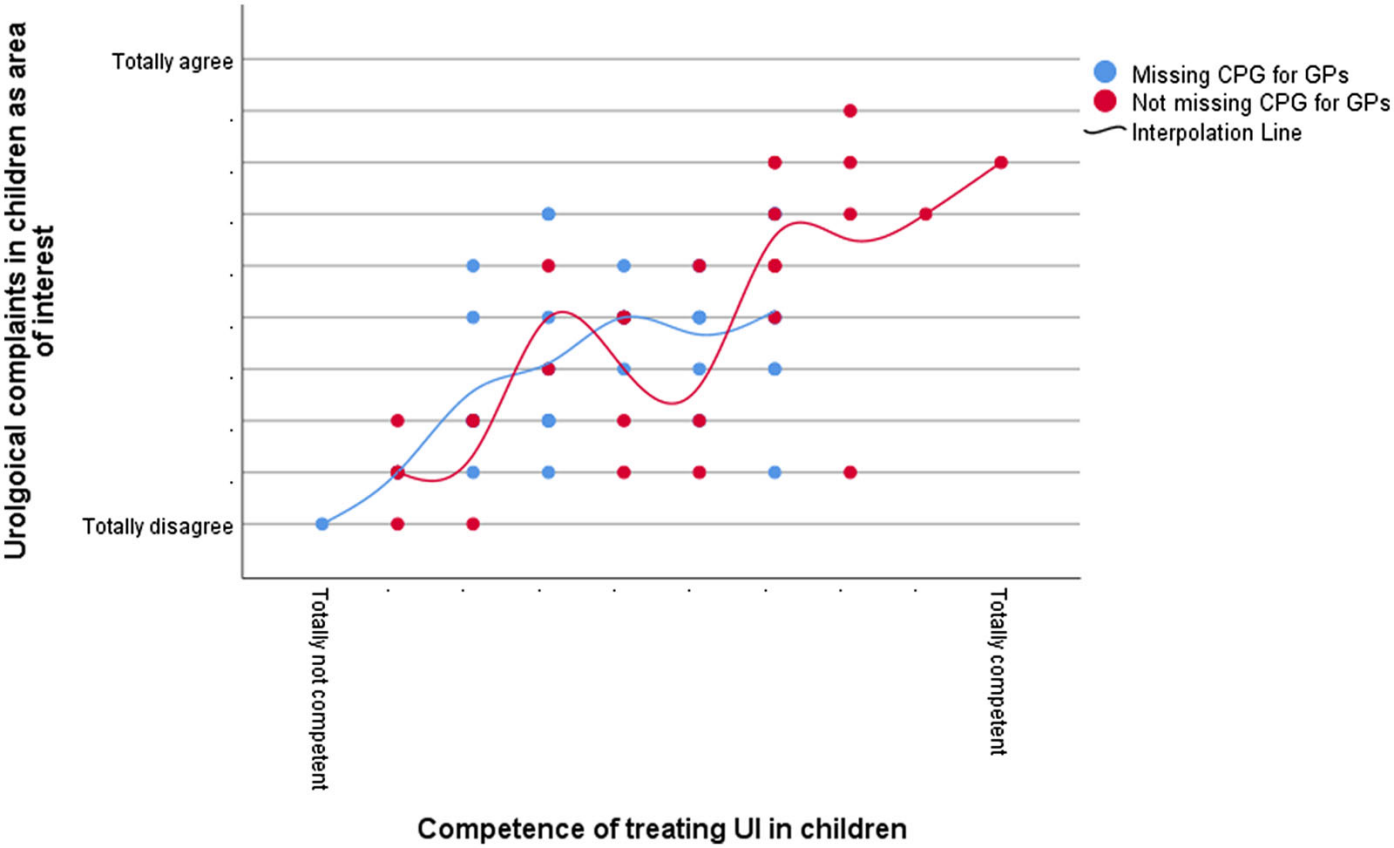
Interest in urological complaints in children by perceived proficiency in treating UI in children. Data are shown for 70 GPs and plotted by whether that GP wanted a clinical practice guideline for daytime UI in children. The *x-* and *y-*axes are shown on 10-point scales. *Abbreviations*: CPG, clinical practice guideline; GPs, general practitioners; UI, urinary incontinence.

Almost half (47.9%) reported having no professional interest in urological complaints in children. Reported interest in urological complaints and feeling competent in treating children with daytime UI were positively related (*r_s_* = 0.664, *p* < 0.001), irrespective of the expressed need for a guideline for daytime UI (Figure 2).

## Discussion

### Main findings

In this study, Dutch GPs reported a lack of competence in treating children with UI and a wish for a clinical practice guideline specific to their needs. The most common justifications for referring a child with daytime UI were the explicit wishes of the children or parents, persistent symptoms despite treatment, the presence of psychosocial factors or other physical complaints. In most instances, children were referred to a paediatrician. Prior to referral, almost three-quarters of GPs obtained a medical history, but few performed any diagnostics or initiated treatment.

### Strengths and limitations

A strength of this study is that the participating GPs had a broad range of experience working in both cities and more rural areas. However, our reliance on data from one hospital in the Netherlands may have generated an unrepresentative sample.

The sampling method may have been both a strength and a weakness. On the one hand, it meant that all participating GPs were involved in caring for children with UI. On the other hand, GPs who treat children with UI will refer fewer children, and we do not know the size of this population or the care they received.

Notably, outcomes could have been biased due to recall by allowing the use of medical records to answer patient-specific questions. By contrast, questions directed at the GP’s general management were unlikely to be affected by recall bias because these reflect their current practice, but could have prompted socially desirable answers.

Finally, it is unsure if the outcomes of this study are generalisable to other European countries with comparable primary care settings, such as Denmark, Norway, England, Italy and Portugal. A study among GPs in Europe showed that GPs in the Netherlands are treating most children themselves instead of referring the child to a specialist [11]. However, this was based on all complaints a child can present with and not specified for UI. Compared to GPs in other countries, the Dutch GP has a broad range of tasks and multiple other responsibilities, which could explain why they need more confidence treating daytime UI, especially because the prevalence of daytime UI is relatively low. Our study shows that almost half of the GPs did not consider urological complaints an area of interest and felt they lacked the skills to treat these children. Previous studies in New Zealand and Australia support this finding [9,10]. The absence of guidelines and uncertainty may explain why GPs refer children with daytime UI directly to hospital for analysis and treatment when some cases could be managed in primary care.

We found that the most common reason for referral was the parents’ explicit wishes. This is in line with an earlier survey among GPs in Europe showing that Dutch GPs are most influenced by patients to make referrals, with 60% of referrals on request of the patient in the Netherlands, compared to 30–40% in countries in South Europe [11]. In case of referral, we found that GPs preferred the more general approach offered by paediatricians when children had problems other than daytime UI, including other physical or psychosocial problems. This is appropriate given the association between such complaints and daytime UI [2,4,6,12–15]. GPs also likely referred children with multiple complaints to a paediatrician because of their specialist knowledge beyond UI and ability to manage all aspects of their care. We expect this to be comparable to other parts of the world, as paediatricians are sometimes considered as the GPs for children.

An essential early step in the treatment of UI is to ensure adequate fluid intake and to complete a voiding diary, which was advised by only about one-third and one-tenth of the GPs, respectively. These approaches have considerable potential as cheap and informative diagnostic aids that can be used by GPs [16,17].

About one-quarter of the GPs obtained only a medical history before referral and performed no diagnostic tests. Although they did report paying attention to toilet position, hygiene, and urinating at set times, they may have yet to learn that these are elements of standard urotherapy. Most GPs indicated that anticholinergics were not suitable for initial therapy, consistent with recent data showing that Australian GPs have a poor knowledge of first-line treatments for daytime UI [10], and compared to other countries, Dutch GPs are reluctant to prescribe medication [11].

### Implications and further research

Suppose we could improve the confidence of GPs in treating daytime UI in children. In that case many uncomplicated cases could be routinely managed in primary care, thereby reducing healthcare costs and demands on hospital care. To achieve this, we should educate GPs about urotherapy (avoiding holding manoeuvres, proper toilet posture, normalisation of fluid intake and timed voiding) [8]. This could be supported by a new GP guideline, in which the basic assessment with simple diagnostic tests and standard urotherapy are explained.

Special attention should be given to voiding diaries that can easily reveal the frequency of UI and the pattern of UI. GPs could use both aspects to advise parents on how to solve the problem themselves. This also ensures that the therapeutic process begins with the active involvement of children and parents.

## Conclusion

This research offers valuable insights in how Dutch GPs assess children with daytime UI. Most GPs do not treat these children but refer them to a paediatrician, and almost half of the GPs feel incompetent to treat children with DUI. Developing a GP guideline for this topic could help prevent unnecessary referrals to hospital, supporting the principle of the right care being delivered in the right place.

## Supplementary Material

Supplemental Material

## Appendix A. Questionnaire management of children with daytime urinary incontinence by general practitioners

The first seven questions are about the child you referred. Your patient’s name and date of birth can be found in the cover letter. This form contains only the patient code. You will find nine more general questions on the back of the questionnaire. Thank you very much for your effort!

I complete this questionnaire for:

□ Myself □ Someone else (for example acting general practitioner)

Patient-specific questions when referring a child in connection with urinary incontinence

1. Which of the following complaints did you request? (Multiple answers possible.)
  □ None □ Defaecation pattern □ Primary or secondary urinary incontinence
  □ Micturition habits □ Nocturnal enuresis
  □ Pain □ Otherwise, namely……………
2. Which diagnostics have you already used on this child? (Multiple answers possible.)
  □ None □ Physical examination □ Genital investigation
  □ Urine test (dipstick or microscopy) □ Urine culture □ Ultrasound kidneys
  □ Voiding diary incl. fluid intake □ Otherwise, namely………………………………………
3. What lifestyle advice did you give? (Multiple answers possible.)
  □ None □ Sufficient fluid intake □ High fibre food □ Adequate toilet posture and hygiene
  □ Voiding at set times
  □ Otherwise, namely………………………………………
4. What drug therapy have you tried? (Multiple answers possible.)
  □ None □ Anticholinergics □ Laxatives □ Otherwise, namely………………………………………
5. Have you tried other therapeutic options? If yes which one? (Multiple answers possible.)
  □ None □ Pelvic floor therapy □ Otherwise, namely ……………………………………………………
6. How did this child’s referral come about? (Multiple answers possible.)
  □ Explicit wish patient/parents
  □ Too little experience/knowledge
  □ Persistent symptoms
  □ Other ……………………………………………………
7. On what did you base your choice for referral to a urologist, paediatrician, or dry bed and pelvic centre for this child?…………………………………………………

***If you refer multiple children, and therefore receive multiple questionnaires, you only need to complete the questions below once.***

*General questions when referring children with urinary incontinence*

1. What is your policy for children with daytime urinary incontinence? (Multiple answers possible).
  □ diagnose and start treatment myself
  □ I refer to a (paediatric) urologist for diagnosis and treatment
  □ I refer to a paediatrician for diagnosis and treatment
  □ I refer to a paediatric pelvic physiotherapist
  □ Otherwise, namely ……………………………………
2. When referring a child to the hospital for urinary incontinence: In what proportion do you choose the urologist or the paediatrician:
  Always urologist ○––○––○––○––○––○––○––○––○––○ Always paediatrician
3. Treatment of urinary incontinence in children with anticholinergics belongs in primary care:
  Totally disagree ○––○––○––○––○––○––○––○––○––○ Totally agree

General questions

1. Since when did you become a general practitioner (year)? ………………………………………………………
2. I consider urological complaints in children as my area of interest:
  Totally disagree ○––○––○––○––○––○––○––○––○–○– Totally agree
3. How skilled do you feel in treating urinary incontinence in children?
  Completely not ○––○––○––○––○––○––○––○––○––○ Maximum
4. Do you consult other guidelines, for example from trade unions, if a child with urinary incontinence comes to your consultation hour? If yes which one? ………………
5. Are you missing an NHG standard for daytime urinary incontinence in children? □ Yes □ No
