# Supplementary material for: Managing children with daytime urinary incontinence: a survey of Dutch general practitioners
Source: Eur J Gen Pract. 2023 Apr 25;29(1):2149731. doi: 10.1080/13814788.2022.2149731 (PMC10132240; doi:10.1080/13814788.2022.2149731)
Supplement: Supplemental Material [file IGEN_A_2149731_SM3921.docx]

**Appendix 1: Questionnaire Management of children with daytime urinary incontinence by general practitioners**

Patientcode: *

The first seven questions are about the child you referred. Your patient's name and date of birth can be found in the cover letter. This form contains only the patient code. You will find nine more general questions on the back of the questionnaire. Thank you very much for your effort!

I complete this questionnaire for:

□ Myself □ Someone else (for example acting general practitioner)

Patient-specific questions when referring a child in connection with urinary incontinence

1. Which of the following complaints did you request? (Multiple answers possible.)

□ None □ Defecation pattern □ Primary or secondary urinary incontinence

□ Micturition habits □ Nocturnal enuresis

□ Pain □ Otherwise, namely………………………………………………………

1. Which diagnostics have you already used on this child? (Multiple answers possible.)

□ None □ Physical examination □ Genital investigation

□ Urine test (dipstick or microscopy) □ Urine culture □ Ultrasound kidneys

□ Voiding diary incl. fluid intake □ Otherwise, namely……………………

…………………………………………………………………………………………

1. What lifestyle advice did you give? (Multiple answers possible.)

□ None □ Sufficient fluid intake □ High fibre food □ Adequate toilet posture and hygiene □ Voiding at set times □ Otherwise, namely…………………………

…………………………………………………………………………………………

1. What drug therapy have you tried? (Multiple answers possible.)

□ None □ Anticholinergics □ Laxatives

□ Otherwise, namely…………………………………………………………………

1. Have you tried other therapeutic options? If yes which one? (Multiple answers possible.)

□ None □ Pelvic floor therapy □ Otherwise, namely ……………………………

…………………………………………………………………………………………

1. How did this child's referral come about? (Multiple answers possible.)

□ Explicit wish patient/parents

□ Too little experience/knowledge

□ Persistent symptoms

□ Other ……………………………………………………………………

1. On what did you base your choice for referral to a urologist, paediatrician, or dry bed and pelvic centre for this child?

………………………………………………………………………………………………………………………………………………………………………………………………………………………………………………………………………………

***If you refer multiple children, and therefore receive multiple questionnaires, you only need to complete the questions below once.***

General questions when referring children with urinary incontinence

1. What is your policy for children with daytime urinary incontinence? (Multiple answers possible).

□ I diagnose and start treatment myself

□ I refer to a (paediatric) urologist for diagnosis and treatment

□ I refer to a paediatrician for diagnosis and treatment

□ I refer to a paediatric pelvic physiotherapist

□ Otherwise, namely…………………………………………………………………

2. When referring a child to the hospital for urinary incontinence: In what proportion do you choose the urologist or the paediatrician:

Always urologist ○----○----○----○----○----○----○----○----○----○ Always paediatrician

3. Treatment of urinary incontinence in children with anticholinergics belongs in primary care:

Totally disagree ○----○----○----○----○----○----○----○----○----○ Totally agree

General questions

1. Since when did you become a general practitioner (year)? ………………………….
2. I consider urological complaints in children as my area of interest:

Totally disagree ○----○----○----○----○----○----○----○----○----○ Totally agree

1. How skilled do you feel in treating urinary incontinence in children?

Completely not ○----○----○----○----○----○----○----○----○----○ Maximum

1. Do you consult other guidelines, for example from trade unions, if a child with urinary incontinence comes to your consultation hour? If yes which one?

…………………………………………………………………………………………

1. Are you missing an NHG standard for daytime urinary incontinence in children?

□ Yes □ No
